# Exploring cardioprotective potential of esculetin against isoproterenol induced myocardial toxicity in rats: in vivo and in vitro evidence

**DOI:** 10.1186/s40360-021-00510-0

**Published:** 2021-07-15

**Authors:** Chitikela P. Pullaiah, Vinod K. Nelson, Sushma Rayapu, Narasimha Kumar G V, Thyagaraju Kedam

**Affiliations:** 1 Department of Pharmacology, Siddha Central Research Institute, Central Council for Research in Siddha, Ministry of AYUSH, Govt of India, Chennai, 600106 India; 2 Department of Biochemistry and College of Pharmaceutical Sciences, S V University, Tirupati, 517502 India; 3 Department of Pharmacology & Toxicology, National Institute of Pharmaceutical Education and Research (NIPER), Hajipur, 844102 India; 4 Department of Pharmacology, Sri Padmavathi School of Pharmacy, Tirupati, 517503 India; 5 Department of Pharmacology, Dr Anjali Chatterjee Regional Institute of Homeopathy, Kolkata, 700035 India

**Keywords:** Isoproterenol, Oxidative stress, Esculetin, Lysosomes, Antioxidant, Cardioprotection

## Abstract

**Background:**

Esculetin is a natural coumarin derivative from various plants with multiple pharmacological effects. Hence, the present study was undertaken to explore the cardio protective potential of esculetin against isoproterenol induced myocardial toxicity in rats.

**Methods:**

The treatment schedule was fixed for 28 days and the rats were divided into five groups of six each. Rats of group I received the normal saline and served as normal control, group II was received ISO (100 mg/kg body weight) for last two consecutive days of the study and served as disease control. Groups III and IV received esculetin 10 and 20 mg/kg body weight respectively once a day per oral for 28 days along with ISO for last two consecutive days of the study. Cardiac biomarkers such as CK-MB and LDH, membrane bound Na+ /K+ ATPases activity, myocardial lysosomal enzymes activity and tissue antioxidants status were estimated in the heart tissue samples. The histopathological changes in the myocardium were also assessed. Further, DPPH assay was done to evaluate the free radicals scavenging potential of esculetin. Cytoxicity assay, intracellular ROS levels by DCFDA assay and m-RNA expression of TNF-α, IL-6 and NF-κB by quantitative RT-PCR in H9c2 cell lines.

**Results:**

The increased levels of CK-MB, LDH, LPO, myocardial lysosomal enzymes and membrane bound Na+ /K+ ATPase levels by ISO administration was significantly increased with concomitant decrease in tissue antioxidant enzymes such as GSH, Catalase, and SOD. Pre-treatment with esculetin for 28 days has significantly decreased the levels of cardiac bio-markers, lysosomal enzymes, membrane bound Na+ /K+ ATPase levels as well as Lipid peroxides which is in contrary to the ISO group. Amelioration of the antioxidant levels were also found in esculetin treated groups. Histopathological examination of heart reveals that myocardial degeneration, mononuclear cell infiltration was noticed in ISO treated rats, whereas the same was restored with esculetin treatment. In H9C2 cell lines esculetin could effectively reduced intracellular ROS inhibition and m-RNA expression of pro-inflammatory cytokines including TNF-α, IL-6 and NF-κB to prevent apoptosis or cell necrosis.

**Conclusion:**

The study provides the evidence of cardioprotective potentials of esculetin against isoproterenol induced myocardial infarction by antioxidant and myocardial membrane stabilization along with in vitro protection from arsenic induced ROS cell necrosis or apoptosis in H9C2 cells.

## Background

Cardiovascular diseases (CVDs) comprise disorders of the heart and blood vessels and still represent a major cause of death globally. CVDs have shown to be responsible for approximately 17.9 million deaths each year, which accounts for 31% of all deaths worldwide. Among the various CVDs, myocardial infarction (MI) is a major cause of mortality and morbidity across the world. Any blockade in the coronary artery leads to insufficient blood supply to heart, causing the heart muscle that is being supplied by the artery to get infarcted ultimately resulting in ischemic tissue necrosis in addition to other pathological and structural changes. The pathogenesis of MI includes hyperlipidemia, oxidative stress, peroxidation of membrane lipids, and loss of plasma membrane integrity [1].

Isoproterenol (ISO), a synthetic catecholamine and β-adrenergic agonist is well-known to cause severe stress in the myocardium by generating free radicals which in turn stimulates lipid peroxidation and perhaps the major contributing factor for the irreversible damage to the myocardial membrane [2]. ISO upon administration causes increase in heart rate leading to increased oxygen demand, high calcium burden and accumulation besides causing alterations in the morphology and membrane integrity of the myocardium with elevated cAMP levels in the myocardial cells [3]**.**

Induction of myocardial infarction was previously performed by surgical procedures, but it has incidence of morbidity, mortality and animals were prone to pneumothorax infections [4]. Isoproterenol-induced myocardial infarction is simple and non-invasive model, considered as one of the most widely used experimental model to study the beneficial effects of many drugs and cardiac function [2] and it is similar to those pathophysiological changes observed in human myocardial infarction [5].

Natural products have high universal demands due to their claimed advantage in terms of both safety and efficacy against various diseases like MI. Plant based coumarins are low- molecular weight phenolic compounds that has been used for the prevention and treatment of various thromboembolism MI and stroke [6]**.** Esculetin (6, 7- di hydroxyl coumarin) is a natural coumarin derivative isolated from many plants such as *Artemisia capillaries, Citrus limonia, Solanum surretten*ce and *Euphorbia lathyris* [7] with multiple pharmacological & biochemical properties [8]. There is numerous treatment approaches that have been developed to attenuate the risk of myocardial infarction, but most of them fail when translated from the bench to the bedside. Therefore, there is a need to find new drugs which hostile MI as well as be used as suitable therapeutic candidate and also can be translated to clinical use in the future [9].

The present study was intended to investigate whether esculetin can provide protection against free radical generation by ISO and its associated alterations in the membrane bound enzymes and myocardial lysosomal enzyme activities in experimental rats. Further, we also tried to explore the probable mechanism of action of the esculetin for its cardio protection in H9C2 cell lines.

### Experimental procedures

#### Chemicals

Isoproterenol (CAS Number 5984-95-2), and Esculetin (CAS Number 305–01-1) was purchased from Sigma Aldrich. Co, St. Louis, USA. All the chemicals used in the present study were of analytical grade and indigenous.

### In vitro studies

#### 2, 2-Diphenyl-1-picrylhydrazyl (DPPH) radical scavenging activity

The antioxidant activity of esculetin was measured using DPPH radical scavenger assay in triplicate. Concentration of 5, 25, 50 and 100 μM/mL were used along with 10 μM of ascorbic acid as reference standard. The test were then incubated with 50 μL of 0.1 mM DPPH solution and made up to the final volume to 3 mL with methanol. A blank was prepared using DPPH solution and methanol. The reaction mixture was incubated for 30 min at room temperature in the dark followed by measuring absorbance at 517 nm [10].

#### H9C2 cell culture

H9C2 myoblast cells from rat’s myocardium were acquired from National Centre for Cell Sciences, Pune, India. The myoblast cells were cultured in Dulbecco’s modified Eagle’s Medium (DMEM) medium supplemented with 10% FBS and 10 ml/l100 × antibiotic–antimycotic solution containing 10,000 units of penicillin and 10 mg/ml streptomycin in 0.9% normal saline in a humidified atmosphere of 95% air and 5% CO_2_ at 37 °C.

#### Measurement of cell viability by MTT assay

**Fig. 1:**
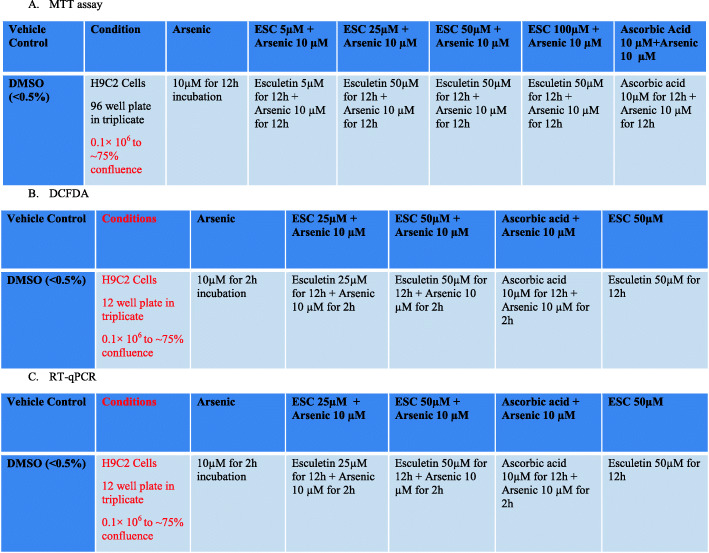
Scheme for experimental design foe H9C2 cell line models **A**. MTT assay protocol. **B**. Protocol for DCFDA assay **C**. Protocol for TNF-α, IL-6 and NF-κB mRNA levels by using RT-qPCR method

The cells were seeded in 96-well culture plates at a density of 7 × 104 cells/well. When the cells reached 80% confluence, they were treated with 1‰ dimethyl sulfoxide (DMSO) solution, and 5, 25 50 and 100 μg/mL of esculetin at 37 °C for 24 h. The cells were then incubated with 3-(4,5-dimethylthiazol-2-yl)-2,5-diphenyltetrazolium bromide (MTT) (0.5 mg/mL) solution for 4 h, and the resulting formazan was solubilized with 150 μL of DMSO for 30 min. The absorbance of each well was measured at 570 nm, and the absorbance of control cells was considered to indicate 100% cell viability [10, 11]. Scheme represented in Fig. 1.

#### Intracellular reactive oxygen species (ROS) measurement

The generation of intracellular reactive oxygen species (ROS) was measured by using the ROS-sensitive fluorescence indicator called Dichlorofluorescin diacetate (DCFH-DA) as per our previous protocol. H9C2 cells grown in 12 well plates 0.1× 10^6^ to ~ 75% confluence were treated in triplicate. The cells were treated with esculetin at 25 and 50 μM/mL concentrations for 12 h. After incubation, 10 μM arsenic were added to esculetin treated wells and incubated for 2 h at 37 °C. All the wells including control were washed with PBS and incubated with 20 μM DCFH-DA for 30 min at 37 °C in the dark. After, Cells were washed, and analyzed by flow cytometer. The florescence intensity was calculated using the FAC Suite software [10]. Scheme represented in Fig. 1.

### RNA isolation, cDNA synthesis and qPCR to assess mRNA expression of TNF-α, IL-6, NF-κb

H9C2 cells grown in 12 well plates 0.1× 10^6^ to ~ 75% confluence were treated in triplicate. After arsenic and esculetin treatment, the total RNA was isolated from H9C2 cells by using Trizol reagent (Thermo Fisher Scientific, Inc.). The isolated RNA was quantified by using a nano-drop spectrophotometer and complementary DNA (cDNA) was synthesized from 1 μg of RNA was used for reverse transcription reaction using the iScript cDNA synthesis kit (Bio-Rad, Hercules, CA, USA). Reverse transcription quantitative polymerase chain reaction (RT-qPCR) was performed by using the SYBR green reagent according to the manufacturer’s protocol (MilliporeSigma).

The primer sequences used for qPCR were as follows: TNF-α forward, 5′ GAACTGGCAGAAGAGGCACT-3′ and reverse, 5′-GGTCTGGGCCATAGAACTGA-3′; IL-6 forward, 5′-CCGGAGAGGAGACTTCACAG-3′ and reverse, 5′-CAG AATTGCCATTGCACA-3′; NF-κB forward 5′-CCCACACTATGGATTTCCTACTTATGG’-3 and reverse 5′ CCAGCAGCATCTTCACGTCTC-3′. RT-qPCR reactions were performed under the conditions like, 50 °C for 35 min, 85 °C for 12 min, followed by 60 cycles of 95 °C for 23 s and 60 °C for 1.5 min. The selected gene expression level was normalized to glyceraldehyde-3-phosphate dehydrogenase (GAPDH) forward, 5′ CTTTGGTATCGTGGAAGG ACTC-3′ and reverse, 5′ GTAGAGGCAGGGATGATGTTCT-3′ as internal loading control [12, 13]. Scheme represented in Fig. 1.

### In vivo study

#### Animals

Male wistar rats weighed between 230 and 280 g were used in this study. Rats were housed under standard conditions and fed with standard pellet with drinking water ad libitum. The animals were kept in polypropylene cages and maintained at a room temperature of 25 ± 2 °C with 55 ± 5% relative humidity and 12 h light/dark cycle. The study was carried out in compliance with the ARRIVE (Animals in Research: Reporting In Vivo Experiments) guidelines (“Guide for the Care and Use of Laboratory Animals” (Institute of Laboratory Animal Resources, National Academic Press 1996; NIH publication number #85–23, revised 1996). All experimental procedures and methods were approved by the Institutional Animal Ethical Committee (IAEC), Sri Padmavathi School of Pharmacy, constitute as per the directions of the Committee for the Purpose of Control and Supervision of Experiments on Animals (CPCSEA), India.

#### Induction of myocardial infarction

Myocardial infarction was induced by dissolving isoproterenol (100 mg/kg) in normal saline and injected subcutaneously to rats for last two consecutive days of the experimental schedule [14].

#### Experimental schedule

The treatment schedule was fixed for 28 days and the 24 rats were divided into four groups of six each. Same timing preferable morning 10 am to 10:30 a m was maintained while dosing every day.

**Table:** 

Group 1	Normal saline serve as control
Group 2	Isoproterenol 100 mg/kg body weight dissolved in 1 mL of normal saline and administered by subcutaneously for last two consecutive days of the study and serve as disease control
Group 3	Esculetin 10 mg/kg body weight dissolved in 1 mL normal saline once daily oral and serve as test group at low dose
Group 4	Esculetin 20 mg/kg body weight dissolved in 1 mL normal saline once daily oral and serve as test group at high dose

#### Blood sample collection and analysis

At the end of treatment blood was collected from retro orbital plexus by anesthetizing the rats with thiopental sodium (35 mg/kg body weight, intra peritoneal) [15] and serum was separated by centrifugation at 2000 rpm. Serum was used to analyze various biochemical parameters such as determinations of cardiac biomarkers lactate dehydrogenize (LDH), and creatinine kinase MB (CK-MB) by using commercial diagnostic kits (Agappe Pvt. Ltd., Kerala, India).

#### Na^+^ /K^+^ ATPase activity of myocardial membrane

The myocardial membrane Na^+^ /K^+^ ATPases activity was determined according to procedure done by Periyathambi and Ponnian 2007. The incubation mixture contained 10 mM of Tris buffer, 20 mM of potassium chloride, 125 mM of sodium chloride, 1 mM of EDTA and 3 mM of ATP. To the incubation mixture, the reaction was initiated by the addition of 0.2 mL of tissue homogenate and the contents were incubated at 37 °C for 15 min. To stop the reaction of 10% trichloro acetic acid (TCA) was added. The tubes were centrifuged and supernatant was used for the estimation of liberated P_i_. 1.0 mL of supernatant was made up to 4.3 mL with distilled water and added 1.0 ml 3 mM of ammonium molybdate reagent. The tubes were incubated at room temperature for 10 min, and later 0.4 ml of amino naptholsulphonic acid reagent was added to develop the color and the P_i_ released recorded using a standard P_i_graph [16].

#### Preparation of lysosomal sub cellular fractions

Lysosomal subcellular fractions were isolated according to the method of Venkatachalem et al.*,*2003. The heart tissue sample was cut open and placed in isotonic saline to remove the blood. Then the heart tissue was rinsed in ice cold 0.25 M sucrose, blotted, weighed and minced. The enzyme extracts were prepared by homogenizing the tissue samples in 0.25 M sucrose at 4 °C. The portion of the homogenate was subjected to differential centrifugation, and the different fractions were separated as follows: structural proteins, nucleus, and cell debris at 600×g for 10 min; mitochondria at 5000×g for 10 min; lysosomes at 15,000×g for 10 min. Myocardial sub-fractions were treated with Triton X-100 (final concentration 0.2% v/v) in ice for 15 min prior to the determination of enzymatic activity [17] **.**

The activities of the lysosomal enzymes including β-glucuronidase [18], β-glucosidase and β-galactosidase [19], and acid phosphatase [20] were determined.

#### Determination of tissue antioxidants

At the end of the experimentation hearts were excised from rats and homogenate in 0.1 M Tris buffer (pH 7.4) and the separated homogenates were used for estimation of heart antioxidants like super oxide dismutase (SOD) [21], Reduced glutathione (GSH) [22], Catalase [23] and lipid peroxidation (LPO) [24].

#### Histopathological studies of heart

After removal of myocardial tissue immediately washed with ice cold saline to remove all the blood and fixed in 10% buffered neutral formalin solution. After fixation was complete, tissues were embedded in paraffin and serial sections were cut in to 0.5 μm. Each section was stained with hematoxylin and eosin. The sections were examined under light microscope and histograms were taken.

### Statistical analysis

Results were expressed as mean ± standard error mean multiple comparisons of the significant analysis of variance (ANOVA) followed by the Dennett’s test as post parametric test using computer based fitting program (Prism graph pad 5.0). A *p* value of < 0.05 was considered as statistically significant.

## Results

### DPPH radical scavenging activity of esculetin

**Fig. 2:**
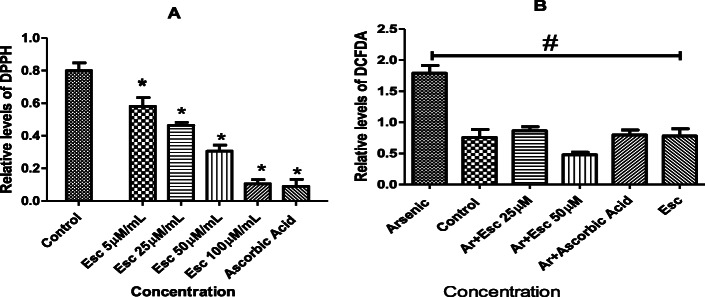
Effect of esculetin on **A**) DPPH radical scavenging potentials of esculetin **B**) Fluorescence intensity of DCFDA, an indicator of ROS level was determined in H9C2 cells. Cells were treated with Esculetin at 25 and 50 25 μM concentrations for 12 h. Each concentration including control (arsenic) tested in triplicate with 75% confluence. Values are presented as the mean ± SEM. * indicates *P* < 0.05 when esculetin and ascorbic acid treatments compared to control. # indicates P < 0.05 when esculetin, ascorbic acid and control treatments wells compared to Arsenic alone treated well

Esculetin exhibited significant radical scavenging activity at all the working concentrations (Fig. 2) nevertheless, the maximum scavenging activity was at 50 μM compared to control.

### Esculetin inhibits intracellular ROS production in H9c2 cell

In the study, we investigated the inhibitory effect of esculetin on arsenic induced ROS generation in H9C2 cells by using DCFH-DA a ROS sensitive non-fluorescent agent (Fig. 2). The DCF stained cells were analyzed by flow cytometer. During incubation period a significant ROS generation is noticed in cells placed with arsenic alone. Whereas cells treated with esculetin at 25 and 50 μg/mL concentrations exhibited dose-dependent inhibitory effects with IC_50_ value of 125 M.

### Effect of esculetin on H9c2 cell viability

**Fig. 3:**
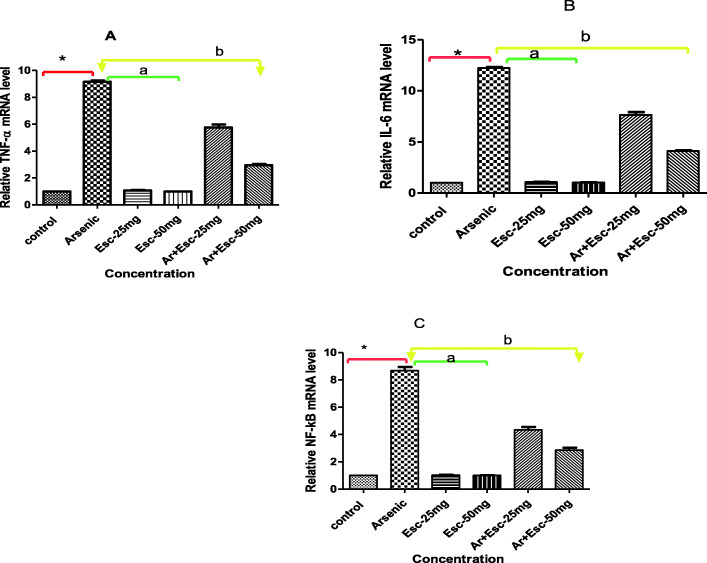
Effect of Esculetin on cell viability in H9C2 cell line. The viability of H9C2 cells treated with different concentrations of esculetin (5, 25, 50, and 100 μM) for 12 h. Each concentration including control (arsenic) tested in triplicate with 75% confluence. Values are presented as the mean ± SEM

MTT assay was used to determine the effect of esculetin on H9C2 cell viability at 5, 25, 50 and 100 μg/mL concentration. After the effective treatment with esculetin H9C2 cells observed as viable with no significant cytotoxicity at all the concentrations (Fig. 3).

### Esculetin attenuates mRNA expression of TNF-α, IL-6, and NF-κB against arsenic induced ROS in H9C2 cells

**Fig. 4:**
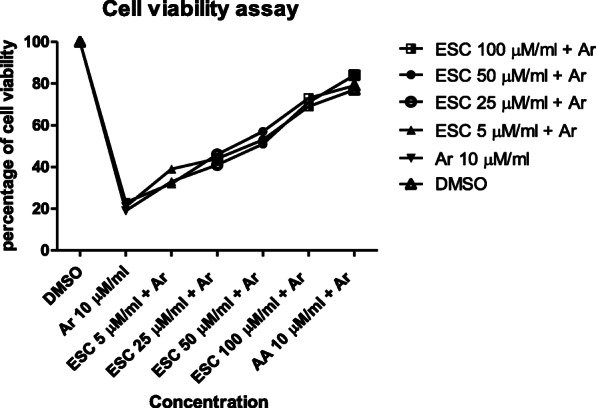
Effect of Esculetin on mRNA expression levels against arsenic induced ROS in H9C2 cells. Cells were treated with Esculetin at 25 and 50 25 μM concentrations for 12 h. Each concentration including control (arsenic) tested in triplicate with 75% confluence. The mRNA levels of pro-inflammatory markers (**A**) TNF-α, (**B**) IL-6 and (**C**) NF-κB were measured by using RT-qPCR. Values are presented as the mean ± SEM. * P < 0.05 when compared control with arsenic a *P* < 0.001 when compared arsenic treatment with esculetin alone. b *P* < 0.005 when compared arsenic treatment with arsenic + esculetin

The mRNA expression of pro-inflammatory cytokines TNF-α, IL-6 and NF-κB were measured using RT-qPCR method against arsenic induced ROS generation in H9C2 cells (Fig. 4). The mRNA expression TNF-α, IL-6 and NF-κB were significantly (*p* ≤ 0.05) elevated, approximately 9 folds compared to control cells. While the cells treated with esculetin at 25 and 50 μg/mL concentration in the presence of arsenic attenuated the expression of mRNA at 4 to 6 folds (p ≤ 0.05) lesser than the control. No differences were observed in the levels of these pro-inflammatory cytokines between the control and esculetin alone groups.

### Effect of esculetin on cardiac biomarkers in ISO induced myocardial toxicity

The alterations in serum heart biomarkers like CK-MB and LDH were tabulated in Table 1. The concentration of CK-MB and LDH were significantly (*p* < 0.05) increased in isoproterenol alone treated rats compared to normal control rats. Animals treated with esculetin brought these cardiac markers near normalcy (p < 0.05) compared to isoproterenol alone treated rats.

**Table 1:** Esculetin effect on CK-MB, LDH and myocardial Na^+^/K^+^ ATPase activity in ISO induced myocardial toxicity

	CK-MB (IU/L)	LDH (IU/L)	Na^+^K^+^ATPases activity (μmol of Pi/mg/h)
Normal control	85.14 ± 4.374	252.0 ± 10.68	3.285 ± 0.2040
Disease control	336.1 ± 23.43^#^	449.6 ± 26.48^#^	1.080 ± 0.158^#^
Esculetin(10 mg/kg)	144.4 ± 6.399^*^	319.9 ± 12.77^*^	2.190 ± 0.099^*^
Esculetin(20 mg/kg)	121.6 ± 7.470^*^	257.9 ± 13.33^*^	2.542 ± 0.303^*^

All values are shown as mean ± SEM and *n* = 6. Data analysis done by one way analysis of variance followed by the Tukey multiple comparison tests. ^#^ *p* < 0.05, indicate disease control compared with normal. * *p* < 0.05, indicate Esculetin treatment compared with disease control group

### Effect of esculetin on membrane bound Na^+^/K^+^ ATPase activity in ISO induced myocardial toxicity

The effect of Esculetin on membrane bound Na^+^/K^+^ ATPase activity has shown in Table 1, significant (*p* < 0.05) decrease of myocardial membrane bound Na^+^/K^+^ ATPase in rats treated with control when compared with normal rats. Whereas, the same Na^+^/K^+^ ATPase significantly (p < 0.05) increased in rats pretreated with esculetin for 4 weeks when compared with control rats.

### Effect of Esculetin on lysosomal membrane destabilization in ISO induced myocardial toxicity

Table 2, shows that alterations of activities of lysosomal hydrolases enzymes like β-glucuronidase, β- galactosidase, β-glucosidase, and acid phosphatase. Activity of these enzymes were significantly (*p* < 0.05) increased in heart tissue homogenates of rats treated with isoproterenol alone when compared with normal rats. Animals pre-treated with esculetin at doses of 10 and 20 mg/kg body weight for 28 days brought these enzyme activities significantly (*p* < 0.05) low and near to normal when compared with isoproterenol alone treated rats.

**Table 2:** Esculetin effect on myocardial lysosomal enzyme’s activity in ISO induced myocardial toxicity

	β-glucuronidase (p-nitro phenol/h/mg protein)	β-glucosidase (p-nitro phenol/h/mg protein)	β-galactosidase (p-nitro phenol/h/mg protein)	Acid phosphatase (p-nitro phenol/h/mg protein)
Normal	0.425 ± 0.003	0.050 ± 0.003	0.042 ± 0.004	0.071 ± 0.008
Disease Control	0.088 ± 0.007^#^	0.114 ± 0.003^#^	0.107 ± 0.007^#^	0.159 ± 0.009^#^
Esculetin (10 mg/kg)	0.536 ± 0.005^*^	0.063 ± 0.005^*^	0.073 ± 0.005^*^	0.084 ± 0.005^*^
Esculetin (20 mg/kg)	0.42 ± 0.001^*^	0.06 ± 0.002^*^	0.062 ± 0.001^*^	0.079 ± 0.003^*^

All values are shown as mean ± SEM and *n* = 6. Data analysis done by one way analysis of variance followed by the Tukey multiple comparison tests. ^#^ *p* < 0.05, indicate disease control compared with normal. * *p* < 0.05, indicate Esculetin compared with disease control group

### Effect of esculetin on tissue antioxidants in ISO induced myocardial toxicity

The changes in heart antioxidants are presented in Table 3. In ISO induced rats, there was a significant (p < 0.05) decrease in GSH, Catalase, SOD and a significant increase in LPO compared to normal control group and pre-treatment with esculetin at doses of 10 and 20 mg/kg, respectively, brought the elevated heart antioxidants near normal.

**Table 3:** Esculetin effect on myocardial tissue antioxidant enzyme’s activity in ISO induced myocardial toxicity

	SOD(U/mg Protein)	CATALASE (μM H202Consumed/mg Protein	GSH (μg of GSH/mg Protein)	LPO (nm of MDA/mg Protein)
Normal	9.248 ± 1.047	7.965 ± 1.217	11.65 ± 1.108	1.693 ± 0.200
Disease Control	1.646 ± 0.516^#^	1.159 ± 0.386^#^	4.027 ± 0.784^#^	3.313 ± 0.407^#^
Esculetin(10 mg/kg)	5.804 ± 0.267^*^	6.484 ± 1.145^*^	7.766 ± 0.384^*^	2.044 ± 0.255^*^
Esculetin(20 mg/kg)	7.241 ± 1.070^*^	9.249 ± 1.856^*^	9.719 ± 0.332^*^	1.661 ± 0.365^*^

All values are shown as mean ± SEM and *n* = 6. Data analysis done by one way analysis of variance followed by the Tukey multiple comparison tests. ^#^ *p* < 0.05, indicate disease control compared with normal. ** *p* < 0.05, indicate Esculetin compared with disease control group

**Fig. 5:**
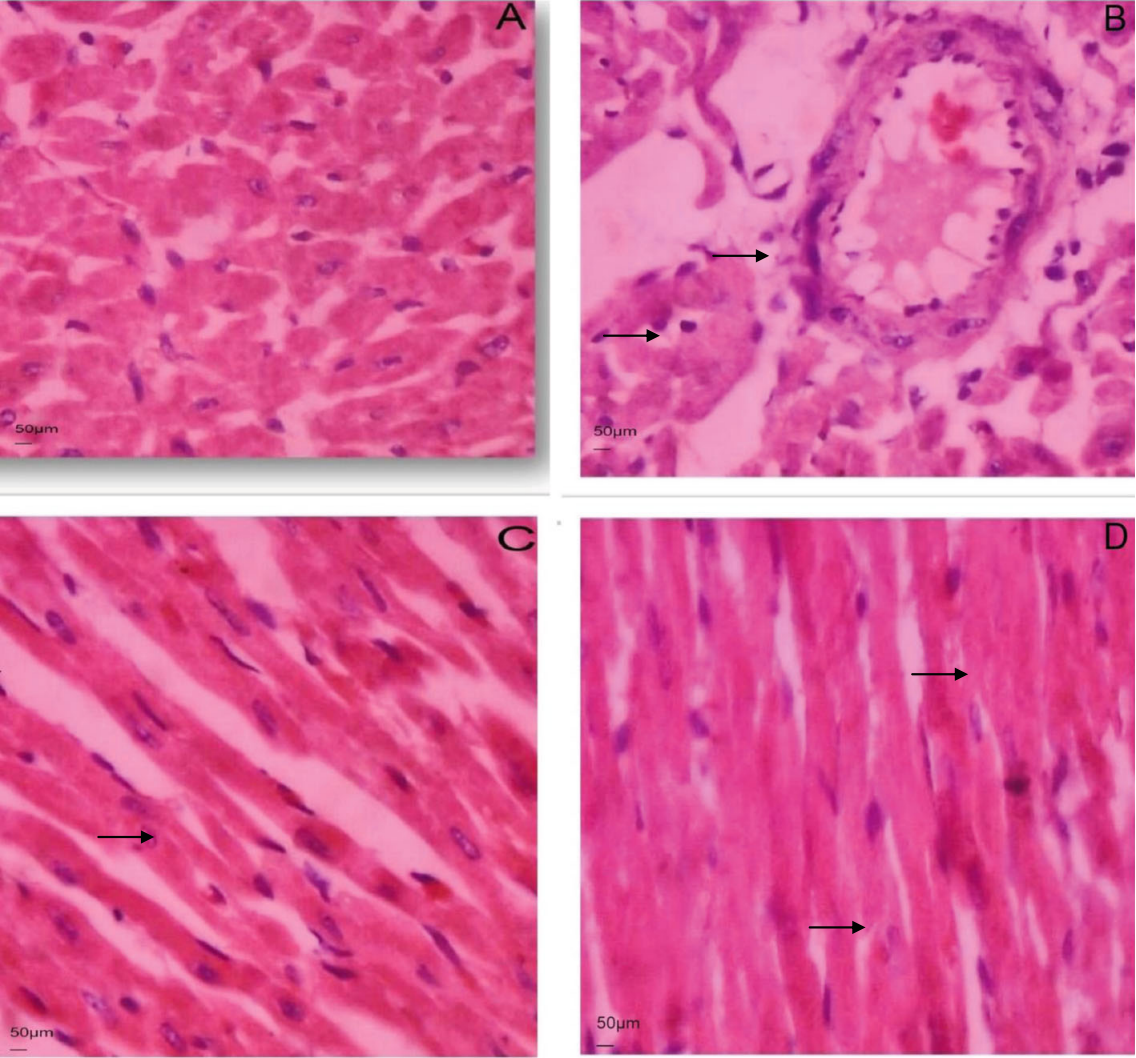
(**A**–**D**): Histopathological study of the heart. **A**): Normal control rat’s heart showing normal texture of cardiac muscle bundles. **B**) ISO induced MI rat’s heart showing area of infarction with inflammatory cell infiltration, cardiac necrosis and splitting of muscle bundles. **C**&**D**) pre-treatment with esculetin showing the prevention of cell infiltration, splitting of muscle bundle by ISO has been prevented

Histopathological observations (Fig. 5) of normal control rat’s heart revealed normal cardiac muscle bundles without any inflammation, whereas myocardium of rats treated with isoproterenol alone has shown a marked inflammatory signs like membrane damage and cellular infiltration along with focal myonecrosis. Rats pre-treated with esculetin at doses of 10 and 20 mg/kg body weight has shown reduction in inflammatory signs and myonecrosis compared to disease control.

## Discussion

The present study validates to reduce the acuteness of isoproterenol induced myocardial infarction by stabilizing the myocardial membrane integrity. The results of experiment bring a new outcome that, pre-treatment with esculetin in experimental rats protects against myocardial infarction. Reactive oxygen species generation by oxidative stress plays an important role in the development of myocardial infarction both experimental and clinically [25].

Antioxidant compounds, especially polyphenol compounds from plants, are capable of counteracting the harmful effects caused by ROS and therefore it can prevent chronic diseases related to oxidative stress [26]. In the present study reveals that, esculetin has shown that direct radical quenching effect in DPPH radical scavenging assay. In addition, it also reduced the intracellular ROS production induced by arsenic [27] in H9C2 cell lines. Treatment with esculetin in H9C2 cells, a noticeable reduction of DCFDA fluorescence intensity was observed this indicates that, esculetin may interact with cellular anti-oxide enzymes. Thus, esculetin has the ability to alleviate free radicals ions both in vitro and in vivo. This was further confirms by tissue antioxidant data of present study.

Anti-oxidant enzymes such as catalase, superoxide dismutase (SOD), glutathione peroxidase and glutathione-S transferase (GSH) are the first line of cellular defense against oxidative injury by superoxide anion radical and H_2_O_2_ before interacting to form the more reactive hydroxyl radical [28]. Auto-oxidation of ISO produces highly cytotoxic free radicals like quinines which in addition with superoxide anion and potent hydroxyl radicals damages the polyunsaturated fatty acids of myocardial membrane [29, 30]. The elevation of these free radicals brings an imbalance between tissue bound cellular scavenging enzymes like SOD, GSH and Catalase and lipid peroxidase [31].

The elevation of free radical including superoxide, hydroxyl ions and hydrogen peroxide ions was the reason for decreasing of tissue bound antioxidant enzymes [32, 33]. It is in line with previous studies, that tissue anti-oxidant enzymes were significantly decreased where the LPO levels were increased in the hearts of ISO alone treated rats [34, 35]. Pre-treatment with esculetin brought the elevated levels of tissue anti-oxidant enzymes like SOD, GSH and Catalase near base line. Our results are agreement with several previous findings in which esculetin is a potential naturally occurring antioxidant both in vitro and in vivo [36, 37].

Probably, the anti-oxidant effect of esculetin could by hydrogen donating ability of esculetin [38], the catechol structure of esculetin contributes the stronger antioxidant activity [36]. The available data of the present study confirms with existing literature that, esculetin has the ability to activate anti-oxidant enzymes or and might combat the excess ROS formed with in the cell [39, 40]**.**

Arsenic one of the major component to induce intra-cellular ROS [27]. Cytokines such as IL-1β, TNF-α and IL-6 are pro-inflammatory markers play a vital role in various inflammatory pathways like JNK, NF-κB and NLRP [41]. NF-κB is a multiple transcription factor that regulates transcription of various genes involved in the pathogenesis of myocardial infarction like TNF-α and IL-6 [42, 43]. These pro-inflammatory cytokines and inflammatory signaling pathways could promote the development of myocardial infarction. Intracellular ROS promotes the production and release of these pro-inflammatory cytokines from cardiomyocytes. TNF-α, IL-1b, and IL-6, are early predictors of organ dysfunction and, causes to activate cardiomyocytes apoptosis [44]. In our study we investigated for the change in the mRNA expression levels of ROS sensitive transcription factor NF-κB along with its pro-inflammatory cytokines TNF-α and IL-6 in esculetin treated H9C2 cells and noticed a dose dependent decline in the mRNA expressions of above said markers. This denotes that esculetin could effectively inhibited the expression of mRNAs of pro-inflammatory cytokines, which are the prominent markers of inflammation and myocardial toxicity, this effect may prevent the H9C2 cell to undergo apoptosis or cell necrosis from arsenic induced ROS. The present findings are in line with earlier research saying esculetin could effectively down regulated the TNF-α, NF-κB and IL-6 levels upon various stimulus including ROS [13, 45, 46].

CK-MB and LDH are indicator biomarker to diagnose the severity of myocardial infarction and number of necrotic cells [47]. These enzymes are present in cardiac muscle, upon the injury release into the blood stream [48]. The available data [49, 50], revels that isoproterenol administration significantly elevates the CK-MB and LDH levels in the serum; in the present study also the same. In the in vitro model [28], esculetin improved the cell viability and reduces the release of LDH, in the same line, pre-treatment with esculetin reduce the ISO induced raise of CK-MB and LDH in rats to protect the myocardium, and it also maintained the cell viability in arsenic induced stress H9C2 cell lines. This implying that esculetin has the potentiality to protect pathological and morphological changes in the rats in a dose-independent manner.

The functioning ability of the membrane bound enzymes are depends on the stability of plasma membrane. Under normal cell physiology Na^+^- K^+^ ATPase will balances the Na^+^ and K^+^ ions across the membrane, upon its disruption may leads to ionic inequality and cell death. Therefore, the determination of membrane bound enzyme activities will indicate any alteration to the membrane physiology under pathological conditions [51]. ISO liberated free radicals bind to the membrane lipids to cause injure, thereby inhibits the membrane associated enzymes function including ATPases [2]. The loss of ATPase activity in the ischemic state may be responsible for causing functional damage and reversible necrotic changes in the involved myocardial cell [52].

Palanivel Karthika et al., has found that, pre-treatment with esculetin has effectively increased the Na^+^- K^+^ ATPase activity, this could be due to the ability of esculetin to protect SH group of Na^+^- K^+^ ATPase enzyme from oxidative damage through inhibition of peroxidation of membrane lipids [53]. It is in line that, the same circumstances were observed in the present study with pre-treatment with esculetin appreciably restored the membrane bound Na^+^- K^+^ ATPase near to normalcy.

Lysosomes are surrounded by phospholipid enriched membranes, and are a potential site for free-radical attack including ISO produces quinines [54]. Therefore, radical ions causing to loss the membrane stability and leads the release of hydrolytic enzymes from its sacs to augments cell necrosis [55, 56]. Hence, significant attention has been raised on the involvement of lysosomal enzymes in myocardial damage. From our previous report it was observed that, administration of the ISO to the rats causes a significant elevation in the activities of the lysosomal enzymes [30]. In the present study same effect observed.

Stabilization of myocardial cell membranes, mainly the lysosomal membranes, may extend the viability of myocardial cells to prevent MI. Pre-treatment with esculetin could effectively inhibit the release of lysosomal enzymes from their sacs, and protects the myocardium from necrosis. This provides the first in vivo evidence that esculetin protects myocardial from ISO-induced injury by lysosomal membrane stabilization.

From in vivo study the biochemical finding that proves the esculetin’s protection in rat’s myocardium was further supported by histopathological examination. Myocardial tissue sections of normal rats depicted clear integrity of the myocardial cell membrane whereas the sections of hearts treated with ISO showed necrosis of muscle fibers with inflammatory cell infiltration, edema and fragmentation of muscle fibers, which indicated involvement of oxidative stress and inflammatory processes. Pre-treatment with esculetin showed the integrity to the structure of myocardium from ISO injury near to the normal myocardium denotes its cardio protective action. The limitation of the study was a clear mechanism of action by which pathway esculetin interfering to reducing the myocardial necrosis has not done.

## Conclusion

Based on the results of this study, it can be concluded that esculetin ameliorates isoproterenol associated pathological features like attenuating the oxidative stress, stabilization of lysosomal, myocardial membrane in the protection of rat’s myocardium. Further, esculetin could effectively down regulates pro-inflammatory markers TNF-α, NF-κB and IL-6 cytokines from the arsenic induced ROS to prevent H9C2 cells to undergo necrosis or death. Understanding the molecular mechanisms involved in the lysosomal enzyme leakage may prove beneficial measures in the prevention of myocardial infarction or toxicity. Thus, esculetin may consider as a potential therapeutic candidate to prevent myocardial toxicity. Further work need to be done in order to establish specific molecular mechanisms by which esculetin prevent myocardial toxicity or infarction and possibility for its clinical application.

## Data Availability

Corresponding author will provide the data used in present work upon the request.

## Abbreviations

CVDs: Cardiovascular diseases
MI: Myocardial infarction
ISO: Isoproterenol
TNF-α: Tumor necrosis factor alfa
IL-6: Including interleukin-6
NF-κB: Nuclear factor kappa B
NLRP3: Nucleotide-binding Leucine-rich repeat Receptors Protein DMEM Dulbecco’s Modified Eagle’s Medium
DMSO: Dimethyl sulfoxide
ROS: Intracellular reactive oxygen species
DCFH-DA: Dichlorofluorescin diacetate
cDNA: complementary DNA
RT-qPCR: Reverse transcription quantitative polymerase chain reaction
LDH: Lactate dehydrogenize
CK-MB: Creatinine kinase MB
TCA: Trichloro acetic acid
SOD: Super oxide dismutase
GSH: Reduced glutathione
LPO: Lipid peroxidation
mRNA: Messenger ribonucleic acid
